# The role of main group elements in shaping the properties of linearly-fused heterohexaarenes

**DOI:** 10.1039/d5cc03235k

**Published:** 2025-09-12

**Authors:** Adrian Espineira-Gutierrez, Ines Caro-Noakes, Min Zhang, Marta Mas-Torrent, Elzbieta Regulska, Carlos Romero-Nieto

**Affiliations:** a Department of Inorganic, Organic and Biochemistry, University of Castilla-La Mancha, Calle Almansa 14 - Edif. Bioincubadora 02008 Albacete Spain carlos.romeronieto@uclm.es; b Instituto Regional de Investigación Científica Aplicada (IRICA), University of Castilla-La Mancha Avenida Camilo José Cela 1 13071 Ciudad Real Spain; c Institut de Ciència de Materials de Barcelona (ICMAB-CSIC), Campus UAB 08193 Cerdanyola del Vallès Spain

## Abstract

Embedding main group elements into the π-scaffold of linearly-fused hexaarenes enables fine-tuning of their optoelectronic properties in solution and in the solid state. Phosphorus leads to the best electron-accepting properties and increases fluorescence quantum yield in solution, silicon maximizes emission in the solid-state, whereas nitrogen leads to the smallest bandgap and promotes stronger intermolecular interactions. These findings offer key insights into structure–property relationships driven by main-group elements, establishing new design principles for the development of improved functional organic materials.

## Abstract 2

Tuning the optoelectronic properties of hexaarenes with main-group elements: phosphorus boosts fluorescence in solution, silicon maximizes solid-state emission, and nitrogen narrows the bandgap.

## Graphical abstract

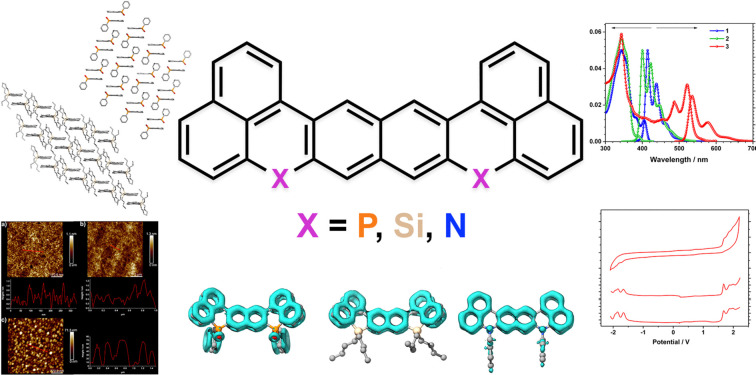

π-Conjugated materials are essential in materials science, with key roles in organic electronics, sensing, and photonics. Their ability to transport charge and absorb or emit light makes them ideal candidates for applications such as OLEDs, OFETs, and solar cells.^1^ Among them, linearly-fused systems stand out for their rigid backbones, extended delocalization, and predictable packing.^2,3^ These features enable high charge mobility and strong, tunable optical responses.^4,5^

Over the past decades, many efforts have focused on modifying linearly-fused frameworks, especially in the development of all-carbon acenes and heteroacenes.^6–8^ For instance, pentacene and its derivatives have been used in OFETs,^9,10^ while nitrogen-containing azaacenes have shown improved stability and n-type behavior.^11^ These studies demonstrated how subtle changes in the molecular backbone can lead to large differences in electronic and solid-state properties. However, new strategies to further diversify these systems remain essential for the next generation of high-performance materials.

A promising, although still underexplored, approach involves inserting main group elements directly into the conjugated skeleton. Unlike external functionalization, such incorporation could affect the geometry, electronic structure, and intermolecular interactions of the molecule. Elements like phosphorus, silicon, and nitrogen vary in size, electronegativity, and orbital participation. As a result, they could reshape conjugation pathways, influence stacking, and tune redox and emission behavior.

Despite several isolated reports,^12–15^ a systematic study that compares how different main group elements alter the properties of linearly fused π-systems is still lacking. Here, we address this gap through a multidisciplinary investigation of linearly-fused hexaarenes containing phosphorus, silicon, or nitrogen (compounds 1, 2 and 3, Scheme 1). We examine their molecular geometry, solid-state organization, electronic delocalization, electrochemical properties and photophysical behavior, uncovering distinct and element-specific effects. Thus, we provide a valuable insight for chemists and materials scientists working on the design of high-performance organic materials. This work advances the fundamental understanding of heteroatom effects in π-conjugated materials and provides a design strategy for future applications in optoelectronics and sensing.

**Scheme 1:**
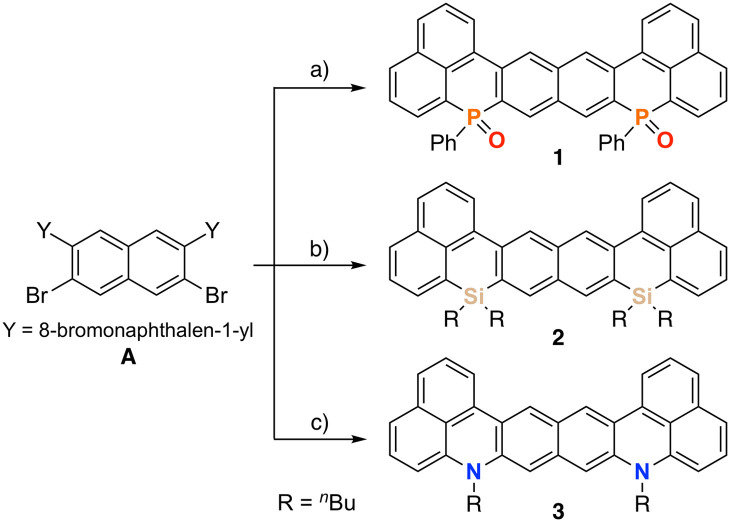
Reaction conditions for the synthesis of hexaarenes 1, 2 and 3. Conditions: (a) 1. ^*t*^BuLi, −80 °C; 2. PhPCl_2_; 3. H_2_O_2_. (b) 1. ^*t*^BuLi, −80 °C; 2. ^*n*^Bu_2_SiCl_2_. (c) Pd_2_(dba)_3_, *R*-BINAP, NaO^*t*^Bu, ^*n*^BuNH_2_.

The synthesis of 1 (Scheme 1) consists of the tetralithiation of the precursor A at low temperature, followed by the double nucleophilic attack to dichlorophenylphosphane.^6*a*^ Analogously, the reaction of the latter tetralithiated derivative with the dichlorodibutylsilane leads to compound 2 with two silicon atoms instead of phosphorus. On the other hand, the preparation of the nitrogen derivative 3 involves the Buchwald-Hartwig reaction between A and *n*-butylamine by using Pd_2_(dba)_3_ and *R*-BINAP as a ligand in the presence of NaO^*t*^Bu. Thus, compounds 1–3 share a common scaffold but differ in the main-group element embedded within the π-framework.^16^

All compounds exhibit outstanding air-, moisture- and photostability; they are soluble in a large list of organic solvents. While 1 is soluble in dichloromethane, chloroform, ethanol, acetonitrile, and tetrahydrofuran, 2 exhibits a relatively improved solubility. It is soluble in pentane, hexane, toluene, diethyl ether, tetrahydrofuran and chlorinated solvents but insoluble in polar protic solvents, including methanol and ethanol. In turn, 3 is soluble in dichloromethane, chloroform and toluene, and shows partial solubility in hexane, tetrahydrofuran, methanol or acetonitrile.

In-depth structural characterization came by single crystal X-ray crystallography. The X-ray structures of 1^6*a*^ and 2 are comparatively displayed in Fig. 1. Unfortunately, 3 containing two nitrogen atoms turned out to be reluctant to crystallize in a wide variety of solvents and techniques (see Table S1), presumably due to its apparent capacity to aggregate and form amorphous particles.

**Fig. 1:**
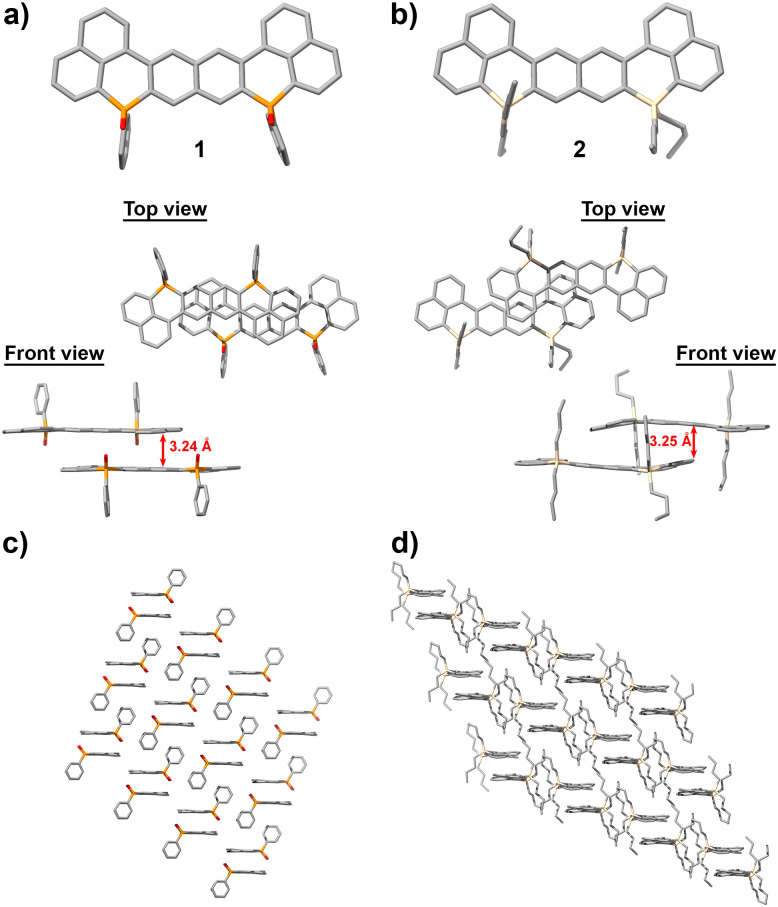
X-ray structures and dimeric packing of (a) compound 1 and (b) compound 2. (c) and (d) Solid-state arrangements of compounds 1 and 2, respectively. Values in red indicate the intermolecular distance in angstrom.

The phosphorus derivative 1 presents a planar framework (Fig. 1a). Its oxygen and phenyl phosphorus substituents lie out of plane. These features promote the formation of slipped dimers with intermolecular distances of 3.24 Å, which are consistent with π–π interactions. In contrast, inserting silicon atoms into linearly-fused molecules leads to slightly bent structures (compound 2, Fig. 1b). The dihedral angles of the main framework are 173 and 165° (Fig. S1). Following the tetrahedral geometry of silicon atoms, the *n*-butyl groups extend over the molecule's surface, leading to an average of 125.1° with respect to the main framework. Nevertheless, compound 2 also forms dimers with intermolecular distances of 3.25 Å. In turn, the 3D arrangement of molecules appears to be governed by the substituents of the heteroatom. While in compound 1 the dimers organize by accommodating the oxygen and phenyl substituents between layers (Fig. 1c), the butyl chains of 2 interact (Fig. 1d), presumably through lipophilic interactions, leading to the 3D arrangement of the dimers (Fig. 1d).

To further explore the solid-state behavior of molecules 1, 2 and 3, we first deposited them onto highly ordered pyrolytic graphite (HOPG) by drop casting and investigated them at the nanometer scale by atomic force microscopy (AFM) (Fig. 2). The results are quite remarkable. While 1 and 2 lead to smooth topologies with rugosities below 1.2 nm, 3 forms rather regular nanospheres with an average diameter of 60 nm. This is consistent with the reluctance of 3 to form single crystals.

**Fig. 2:**
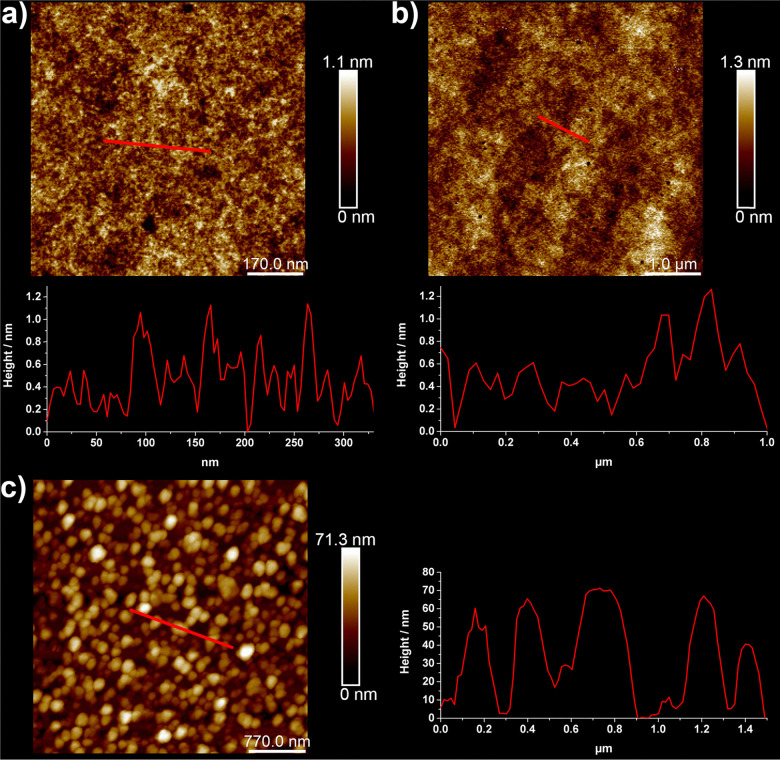
Images obtained from (a) compound 1, (b) compound 2, and (c) compound 3 deposited onto HOPG by atomic force microscopy together with their height profile in nm. Scale bars are: 170 nm for (a), 1 μm for (b) and 770 nm for (c).

To evaluate the optoelectronic properties of compounds 1, 2 and 3, we turned to DFT calculations at the B3LYP/6-311+G(d) level of theory using the PCM model and DCM as solvent. The calculated energy levels and frontier molecular orbitals are shown in Fig. 3, Fig. S2 and Table S4. The electronic distributions of the HOMO and LUMO from 1, 2 and 3 differ substantially depending on the heteroatom embedded in the π-structure (Fig. 3 and Fig. S2). In 1, containing two phosphorus atoms, the heteroelement is involved in the LUMO but not in the HOMO. In turn, in 2, the silicon atom does not participate in either the HOMO or in the LUMO. In contrast, both nitrogen atoms from 3 are included in the HOMO. These differences are reflected in the energy of the frontier molecular orbitals. The energies of the HOMO and LUMO of 1 are −6.05 and −2.42 eV, respectively, providing an optical bandgap of 3.63 eV. The silicon derivative 2, with no participation of the heteroatom into the frontier molecular orbitals, possesses significantly higher relative energies; *i.e.* −5.72 eV for the HOMO and −1.93 eV for the degenerated LUMO and LUMO+1, respectively. The calculated bandgap for 2 is 3.78 eV. This emphasizes the role of the phosphorus atoms in reducing the energy of the frontier molecular orbitals and, thus, the optical bandgap. Finally, theoretical calculations predict for the nitrogen-containing 3 a more pronounced impact of the heteroatoms into the energy of the molecular orbitals. The HOMO of 3 appears as high as −4.90 eV. This is presumably due to an increase in the interelectronic interactions provided by the electron lone pairs of the nitrogen atoms, which is included in the HOMO. In turn, the LUMO of 3 lays at −2.09 eV leading to the smallest bandgap; *i.e.* 2.8 eV.

**Fig. 3:**
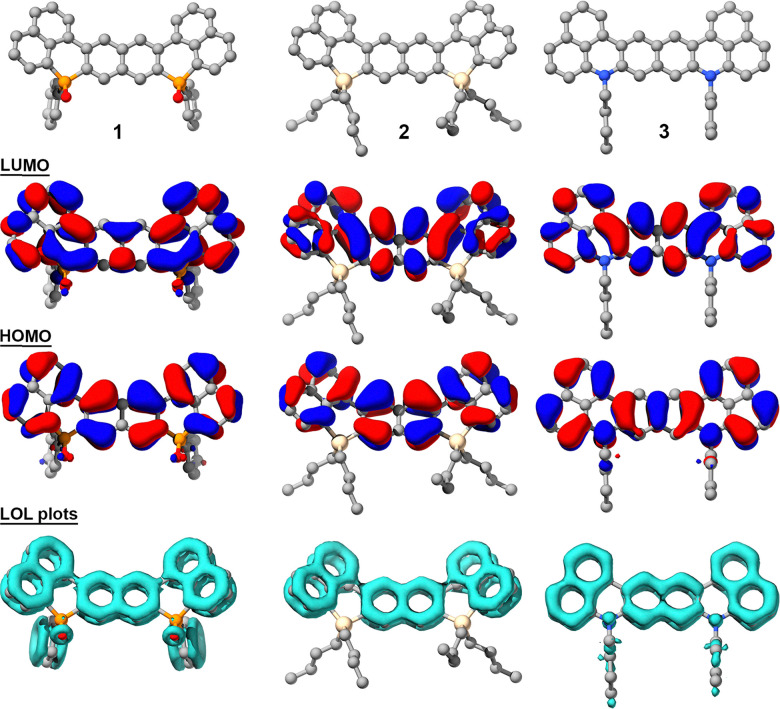
Structures, frontier molecular orbitals and LOL plots of compounds 1, 2, and 3 obtained by DFT calculations.

The localized orbital locator (LOL) plots^17^ further support differences in π-electron distribution influenced by the heteroatoms (Fig. 3). In compound 1, the P=O units and the phenyl phosphorus substituents participate in the π-system. While slight localization appears around the oxygen atoms due to their lone pairs, LOL plots indicate a delocalization of across the P=O fragments, highlighting the role of the P-substituents. Compound 2, which presents two silicon atoms, displays a continuous delocalized electronic π-cloud. However, the tetrahedral silicon atoms do not engage in conjugation but do not interrupt the π-framework *i.e.* the molecule remains silent to the heteroatom. This is consistent with the lack of participation of the silicon atoms in the HOMO and LUMO. In turn, in compound 3, the nitrogen atoms contribute with their lone pair to the π-cloud, resulting in a fully delocalized system. These differences highlight how the electronic nature and geometry of the heteroatom, together with its substituents, modulate π-delocalization within the extended conjugated framework.

To verify the predictions from theoretical calculations, we comparatively investigated the electronic properties of 1, 2 and 3 by electrochemical techniques such as cyclic voltammetry, differential pulse voltammetry and square wave voltammetry (Fig. S4). In 1, the presence of two phosphorus atoms leads to the relatively smallest reduction potentials; it presents two reversible reductions at −1.43 and −1.71 V (Table S5). This is attributed to hyperconjugative effects from the out-of-plane substituents of the phosphorus atoms.^6*a*^ The oxidation process of 1 is located at +1.77 V. The electrochemical properties of the silicon derivative 2 turned rather complex; it exhibits three irreversible oxidations at +1.68, +1.88 and +1.98 V and three irreversible reductions at −1.66, −1.85 and −1.97 V. Thus, the electron donating ability of 2 is slightly better than for the diphosphahexaarene 1. Finally, 3 displays three irreversible oxidations at +0.92, +1.3 and +1.58 V, although no reduction processes could be detected under our experimental conditions (see SI). As predicted by DFT calculations, these values confirm the relatively best electron-donating ability of compound 3.

We further investigated the properties of the linearly-fused derivatives by steady-state spectroscopy (Fig. 4 and Table S6). Compounds 1, 2 and 3 display an absorption maximum at 343 nm, which can be potentially attributed to a transition from the common π-scaffold of all derivatives. However, the most red-shifted absorption maxima depend on the heteroatom embedded in the molecular skeleton. TD-DFT calculations indicate HOMO–LUMO transitions to be responsible for the latter low-energy transitions (see SI, Table S3). While 1 displays an absorption maximum at 405 nm,^6*a*^ the silicon derivative 2 shows a band at 390 nm. The nitrogen derivative 3 presents two absorption maxima at 487 and 522 nm, which are strongly red-shifted in line with the results from theoretical calculations.

**Fig. 4:**
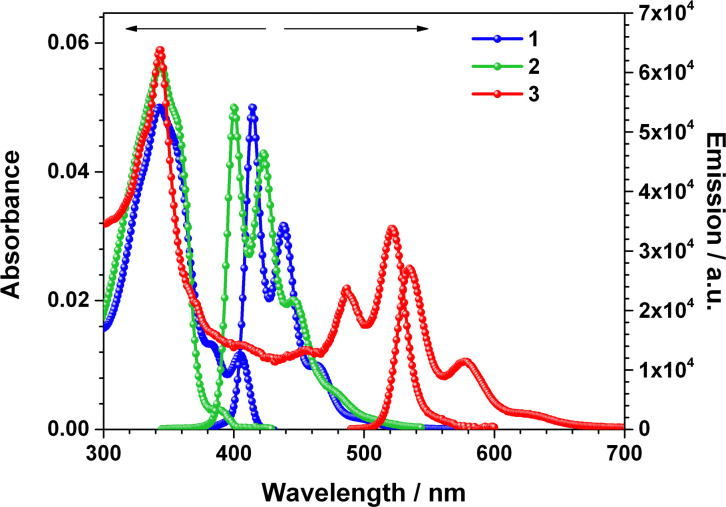
Absorption and emission spectra of compounds 1, 2 and 3 from DCM solutions.

The emission properties were found to be strongly dependent on the heteroatom. While 1 presents three emission maxima at 415, 438 and 463 nm, 2 exhibits three emissions bands that maximize at 400, 423 and 446 nm with a shoulder at 480 nm. Both compounds lead to rather similar color coordinates; *X* = 0.16; *Y* = 0.04. Remarkably, the emission maxima of the nitrogen derivative 3 are red-shifted by 135 nm compared to compound 2 to 535 and 579 nm, with a shoulder at 626 nm. The color coordinates for 3 are *X* = 0.37; *Y* = 0.61, which correspond to a green color. Thus, silicon leads to the most hypsochromically-shifted blue color, followed by phosphorus, and nitrogen leading to a green emission color. The fluorescence decay of compound 1 is monoexponential, with a lifetime of 6.2 ns, whereas 2 exhibits a biexponential decay with relatively longer lifetimes of 9.0 and 18.7 ns (Fig. S7). In contrast, compound 3 presents shorter lifetimes of 1.3 and 3.4 ns, typical of π-conjugated molecules. Accordingly, the incorporation of phosphorus atoms into linearly-fused molecules leads to the highest fluorescence quantum yield in solution (*Φ* = 84%), which is consistent with a monoexponential decay and, thus, the absence of significant non-radiative pathways. This is followed by nitrogen derivative 3 (*Φ* = 74%), while the silicon analogue shows the lowest value (*Φ* = 50%). In the solid state, the scenario differs. Compound 1 emits weakly (*Φ* = 2%) and compound 3 is non-emissive, which is consistent with its pronounced capacity to form assemblies. Remarkably, compound 2 retains high emission in the solid state, with a quantum yield of *Φ* = 55%.

To conclude, the insertion of main-group elements into linearly-fused molecules results in clear differences in their structural and optoelectronic properties. Phosphorus, silicon, and nitrogen, along with their associated substituents, strongly influence the molecular geometry and packing in the solid state. Phosphorus and silicon derivatives form dimers that assemble in the solid state. The nitrogen derivative shows a pronounced tendency to assemble into nano-structures, which was corroborated by AFM. The electronic delocalization is strongly impacted by the heteroelement. Phosphorus and nitrogen contribute to the π-delocalization, whereas silicon remains electronically silent. As a result, phosphorus and silicon derivatives emit in the blue region of the visible spectrum and the nitrogen one in the green. The nitrogen-containing molecule behaves as an electron donor, and the phosphorus and silicon display ambipolar redox character. The variations in the electronic properties are reflected in both solution and solid-state fluorescence properties. Phosphorus leads to the highest fluorescence quantum yields in solution. Notably, the silicon-based compound exhibits high emission efficiency in the solid state, despite modest performance in solution. In contrast, the nitrogen-containing molecule displays a remarkable fluorescence in solution but it is non-emissive in the solid state, consistent with its strong tendency to aggregate. This work represents a detailed and multidisciplinary systematic investigation, contributing to the fundamental understanding of how inserting main-group elements governs structure–property relationships. These insights provide a rational basis for the design of functional π-materials, particularly linearly-fused systems, in applications ranging from optoelectronics to sensing. Future work will explore the incorporation of electron-donating and -withdrawing groups to further modulate the properties of these heterohexaarenes.

## Conflicts of interest

There are no conflicts to declare.

## Supplementary Material

CC-061-D5CC03235K-s001

CC-061-D5CC03235K-s002

## Data Availability

The data supporting this article have been included as part of the SI. All experimental details, spectra and procedures are available in the SI. See DOI: https://doi.org/10.1039/d5cc03235k CCDC 2454905 contains the supplementary crystallographic data for this paper.^18^
