# Supplementary material for: The role of main group elements in shaping the properties of linearly-fused heterohexaarenes
Source: Chem Commun (Camb). 2025 Sep 12;61(80):15590–3. doi: 10.1039/d5cc03235k (PMC12426592; doi:10.1039/d5cc03235k)
Supplement: CC-061-D5CC03235K-s001 [file CC-061-D5CC03235K-s001.pdf]

Supplementary Information (SI) for Chemical Communications.
This journal is © The Royal Society of Chemistry 2025

Supplementary Information for:

The role of main group elements in shaping the properties of linearly-fused heterohexaarenes

Adrian Espineira-Gutierrez,[a,b] Ines Caro-Noakes, [a,b] Min Zhang,[c] Marta Mas-Torrent,[c] Elzbieta Regulska,[a,b] Carlos Romero-Nieto*[a,b]

[a] Department of Inorganic, Organic and Biochemistry University of Castilla-La Mancha, Calle Almansa 14 - Edif. Bioincubadora, 02008, Albacete, Spain.

[b] Instituto Regional de Investigación Científica Aplicada (IRICA), University of Castilla-La Mancha, Av. Camilo José Cela, 1, 13071, Ciudad Real, Spain.

[c] Institut de Ciència de Materiales de Barcelona (ICMAB-CSIC), Campus UAB, 08193, Cerdanyola del Vallès, Spain.

Email: Carlos.RomeroNieto@uclm.es

Table of Contents:

1. General section **2**

2. Experimental details **5**

2.1. Synthetic procedures **5**

2.2. Crystallization conditions for compound 3 .... **7**

3. X-ray structure analysis **8**

4. DFT calculations **10**

5. Electrochemical investigations of compounds 1, 2, and 3 .... **13**

6. Steady-state spectroscopy of compounds 1, 2, and 3 .... **14**

7. NMR data **16**

8. Literature **19**

1. General section

Reactions were carried out in dry glassware and under inert atmosphere of nitrogen using Schlenk techniques. Anhydrous diethyl ether (Et_2O), tetrahydrofuran (THF) and toluene were used directly from a solvent purification system MB SPS-800. Standard solvents such as acetonitrile (MeCN), chloroform ($CHCl_3$), dichloromethane (DCM), ethyl acetate (EtOAc), hexane, isopropanol (*i*-PrOH), methanol (MeOH), pentane and toluene were purchased from commercial suppliers and used as received. Deuterated solvent such as chloroform-d ($CDCl_3$) was purchased from commercial supplier and used as received or dried over molecular sieve. Hydrochloric acid (HCl) (36.5% - 38% aq. solution), magnesium sulfate ($MgSO_4$), *n*-butylamine ($^nBuNH_2$), *n*-dibutyldichlorosilane ($^nBu_2Cl_2Si$), *R*-(+)-BINAP, sodium *tert*-butoxide (NaO^tBu), *tert*-butyllithium (^tBuLi) (1.7 M solution in pentane) and tris(dibenzylidenacetone)dipalladium (0) ($Pd_2(dba)_3$) were purchased from commercial suppliers and used as received. 3,6-Dibromonaphthalene-2,7-diyl bis(trifluoromethanesulfonate) was synthesized following the reported protocols.[S1]

NMR: ^1H, ^{13}C and COSY spectra were recorded at the Edificio Bioincubadora at the Faculty of Pharmacy at the University of Castilla-La Mancha on a 400 MHz Varian Inova NMR spectrometer. Chemical shifts are expressed as parts per million (ppm, δ) and referenced to solvent signals (^1H / ^{13}C): $CDCl_3$ (chloroform-*d*) (7.26 / 77.16 ppm) as internal standard. Signal descriptions include: s = singlet, d = doublet, t = triplet, m = multiplet and br = broad. All coupling constants are absolute values and *J* values are expressed in Hertz (Hz).

Mass spectrometry: HR-ESI spectra were measured by the NUCLEUS analytical service of the University of Salamanca. GC-MS was performed in a GC system 7250 GC/Q-TOF from Agilent Technologies.

X-Ray Crystallography: X-ray crystal structure analyses were measured on Bruker Smart CCD or Bruker Smart APEX instrument using Mo-Kα radiation. Diffraction intensities were corrected for Lorentz and polarization effects. An empirical absorption correction was applied using SADABS[S2] based on the Laue symmetry of reciprocal space. Heavy atom diffractions were solved by direct methods and refined against F2 with the full matrix least square algorithm. Hydrogen atoms were either isotropically refined or calculated. The structures were solved and refined using the SHELXTL[S3] software package. Crystals of **2** (CCDC2454905) were obtained by evaporation from chloroform in the presence of triethylamine.

High-Performance Liquid Chromatography (HPLC): Chromatographic purification was made with an Agilent Infinity II HPLC/UV-Vis model equipped with an Agilent 1290 Infinity II fraction collector, using an Agilent Prep-C18 column (250 mm x 21.2 mm, 10 µm). The isocratic elution was performed using 20 ml/min flow.

Theoretical calculations: Molecular optimizations were carried out at the B3LYP/6-311+G(d) level of theory by using the GAUSSIAN 16 suite of programs.[S4] Solvent effects (DCM) were considered by using the polarization continuum model (PCM).

Electrochemistry: Voltammograms (cyclic voltammetry, differential pulse voltammetry, and square wave voltammetry) were recorded using a Metrohm Autolab PGSTAT101 potentiostat/galvanostat from acetonitrile solutions using tetrabutylammonium hexafluorophosphate as electrolyte, glassy carbon as working electrode, Pt wire as counter electrode, and Ag wire as pseudo-reference electrode. Scan rate is 100 mV s^{-1}. The curves were calibrated using ferrocene as internal standard ($E_{1/2}$ = 0.54 V vs. SCE).

Steady-state spectroscopy: Absorption and emission spectra were recorded from DCM solutions using an Agilent Cary 8454 UV-Vis spectrophotometer and Edinburgh Instruments FS5 spectrofluorometer, respectively.

Fluorescence quantum yields Φ: Quantum yields in solution were measured using quinine sulfate in 0.1 M sulfuric acid as a reference (Φ = 0.54 according to the literature[S5]) from five dilutions and using the formula:

$\Phi_x = \Phi_{st}\ (Grad_x/Grad_{st})\ (n_x^2/n_{st}^2)$

Φ_x: quantum yield of sample X

Φ_{st}: quantum yield of the reference

$Grad_x$: Gradient from the plot of integrated fluorescence intensity vs. absorbance of the sample X

$Grad_{st}$: Gradient from the plot of integrated fluorescence intensity vs. absorbance of the reference

n_x: refractive index from the solvent employed with the sample X

n_{st}: refractive index from the solvent employed with the reference

Detailed procedure: The compound was dissolved in the solvent specified in the main text, and the absorbance at the selected excitation wavelength was adjusted to be below 0.1 to ensure measurements were within the linear range of the Lambert–Beer law. The emission spectrum was then recorded using the selected excitation wavelength. This procedure was repeated using four additional dilutions of arbitrary concentrations, always with the same excitation wavelength.

The area under each fluorescence emission spectrum was integrated, yielding five values that were then plotted against the corresponding absorbance values at the excitation wavelength. Linear regression of this data provided a slope ($Grad_x$) for the sample.

An identical procedure was followed using quinine sulfate dissolved in 0.1 M H_2SO_4 (aq) as the reference standard, yielding a slope ($Grad_{st}$). The refractive index values (n_x and n_{st}) were taken as 1.424 for DCM and 1.333 for water, respectively. The reference fluorescence quantum yield

(Φ_{st}) was set to 0.54. By inserting all values into the equation illustrated above, we obtained the quantum yield of each compound.

The fluorescence quantum yield in the solid state was obtained with an integrating sphere from Edinburgh Instruments.

Fluorescence lifetimes τ: The fluorescence decays were recorded with an Edinburgh Instruments FLS920 single photon counting system with an EPLED-360. Fluorescence lifetimes were acquired by an exponential fit according to the least mean square with commercially available software Edinburgh Instruments F900.

Atomic Force Microscope: The AFM images were registered with a Bruker MultiMode 8-HR microscope using PeakForce tapping mode and a cantilever of a nominal spring constant of 0.4 N/m.

2. Experimental details

2.1. Synthetic procedures

Compound 2

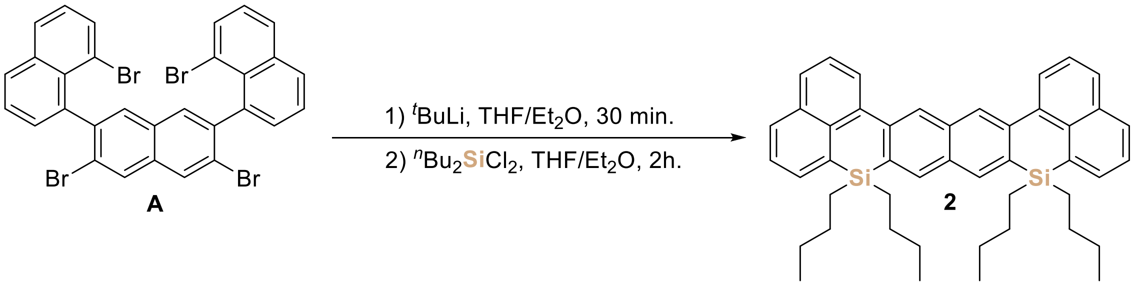

A (1.0 eq, 71,8 µmol, 50 mg) was dissolved in 4.4 mL of a mixture of dry Et_2O:THF (1:1) in a flame-dried Schlenk tube. The mixture was cooled down to –80 °C during 15 minutes. After that, ^{t}BuLi (4.05 eq, 291 µmol, 0.17 mL, 1.7 M solution in pentane) was added dropwise during 1 minute, and the mixture was stirred for 30 minutes at the same temperature. Then, $^n Bu_2SiCl_2$ (2.1 eq, 150.8 µmol, 36.1 µL) was added dropwise. Once the addition was finished, the mixture was quickly set in a preheated bath at 50 °C and stirred for 5 minutes, followed by stirring at room temperature for 2 hours. When the reaction was finished, it was quenched with 10 mL of H_2O, and the aqueous layer was extracted with Et_2O (3x5 mL). The combined organic layers were dried over $MgSO_4$, filtered, and the solvent was removed under reduced pressure. The crude was purified by reverse phase HPLC using isocratic conditions with MeCN/^{i}PrOH (50:50) followed by a short filtration through SiO_2 using toluene:hexane (2:8) to obtain a white solid. Yield: 49% (23.2 mg, 35.1 µmol).

^{1}H NMR (400 MHz, Chloroform-*d*): δ 8.71 (s, 2H), 8.52 (d, *J* = 7.7 Hz, 2H), 8.25 (s, 2H), 7.97 (dd, *J* = 8.4, 1.4 Hz, 2H), 7.91 (d, *J* = 8.2 Hz, 2H), 7.87 (dd, *J* = 6.6, 1.5 Hz, 2H), 7.65 (t, *J* = 7.8 Hz, 2H), 7.58 (dd, *J* = 8.1, 6.6 Hz, 2H), 1.29 – 1.22 (m, 16H), 1.12 – 1.08 (m, 8H), 0.76 (t, *J* = 7.1 Hz, 12H). **^{13}C{^{1}H} NMR** (101 MHz, Chloroform-*d*): δ 140.8 (2C), 135.6 (C), 135.4 (2C), 135.1 (2C), 134.2 (2C), 134.1 (2C), 133.4 (2C), 132.1 (2C), 131.4 (2C), 130.6 (2C), 130.3 (C), 129.9 (2C), 126.4 (2C), 126.1 (2C), 125.4 (2C), 125.1 (2C), 26.6 (4C), 26.1 (4C), 14.9 (4C), 13.8 (4C). **HRMS** (ESI+) calcd. for $[M+H]^+$ $C_{46}H_{53}Si_2^+$ 661.3686, found 661.3664. **EA** calculated for ($C_{46}H_{52}Si_2$): C, 83.57; H, 7.93. Found: C, 83.17; H, 7.75.

Compound 3

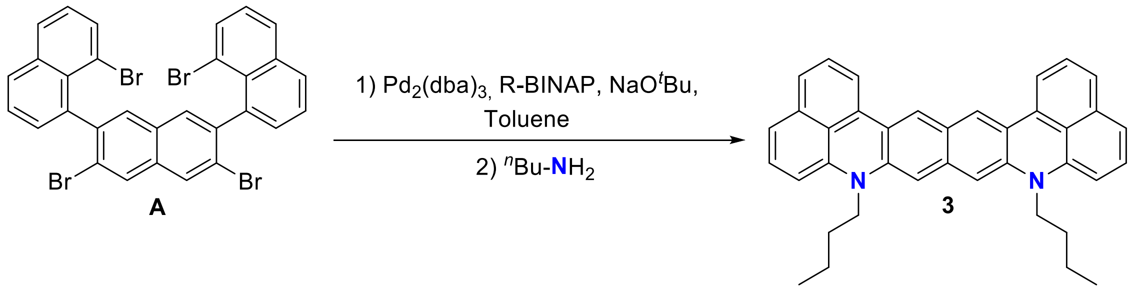

A (1.0 eq, 143.6 µmol, 100 mg), $Pd_2(dba)_3$ (0.06 eq, 8.62 µmol, 10.5 mg), *R*-BINAP (0.24 eq, 34.5 µmol, 21.5 mg) and NaO^tBu (4.4 eq, 632.1 µmol, 62.6 mg) were added to a flame-dried two-necked round-bottom flask connected to a condenser. After that, degassed dry toluene (2 mL) was added to the flask and the mixture was stirred at room temperature for 20 minutes. Then, *n*-butylamine (2.05 eq, 294.5 µmol, 29 µL) was added to the mixture and set in a preheated bath at 90 °C. The mixture was stirred for 24 hours and monitored by ^{1}H-NMR and TLC. When the reaction was completed, it was allowed to reach room temperature and 10 mL of H_2O were added. The pH was neutralized using HCl 3 M. Then, the layers were separated, and the aqueous layer was extracted with $CHCl_3$ (4x5 mL). The combined organic layers were dried over $MgSO_4$, filtered, and the solvent was removed under reduced pressure. The crude was washed several times with Et_2O until the pure product was obtained as a red-brownish solid. Yield: 40% (24.9 mg, 47.9 µmol).

^{1}H NMR (400 MHz, Chloroform-*d*): δ 8.28 (s, 2H), 7.90 (d, *J* = 7.4 Hz, 2H), 7.55 (d, *J* = 8.0 Hz, 2H), 7.46 (t, *J* = 7.7 Hz, 2H), 7.32 (t, *J* = 7.9 Hz, 2H), 7.17 (d, *J* = 8.1 Hz, 2H), 7.02 (s, 2H), 6.60 (d, *J* = 7.8 Hz, 2H), 4.00 (t, *J* = 8.4 Hz, 4H), 1.93 (p, *J* = 7.8 Hz, 4H), 1.67 (h, *J* = 7.4 Hz, 4H), 1.16 (t, *J* = 7.3 Hz, 6H). **^{13}C{^{1}H}NMR** (101 MHz, Chloroform-*d*): δ 139.57 (2C), 139.46 (2C), 136.58 (C), 135.55 (2C), 129.85 (2C), 127.38 (2C), 127.14 (2C), 125.38 (2C), 124.61 (C), 123.10 (2C), 122.16 (2C), 121.53 (2C), 117.45 (2C), 115.06 (2C), 105.90 (2C), 103.83 (2C), 46.46 (2C), 27.08 (2C), 20.48 (2C), 14.16 (2C). **HRMS** (ESI+) calcd. for $[M+H]^+$ $C_{38}H_{35}N_2^+$ 519.2795, found 519.2787. **EA** calculated for ($C_{38}H_{34}N_2$): C, 87.99; H, 6.61; N, 5.40. Found: C, 88.23; H, 6.57; N, 5.02.

2.2. Crystallization conditions for compound 3

Table S1. Conditions employed for the crystallization attempts of **3**.

**Solvent system**	**Method**	**Solvent system**	**Method**
DCM	Slow evaporation	$CHCl_3$/ Acetone	Diffusion
$CHCl_3$	Slow evaporation	TCE	Slow Evaporation
DCM/Pentane	Vapor Diffusion	TCE/Hexane	Vapor Diffusion
DCM/Hexane	Diffusion	TCE/MeOH	Diffusion
DCM/MeOH	Diffusion	THF	Slow Evaporation
DCM/EtOH	Diffusion	THF/Pentane	Diffusion
DCM/Et_2O	Diffusion	THF/Pentane	Vapor Diffusion
DCM/Et_2O	Vapor Diffusion	THF/Acetone	Vapor Diffusion
DCM/Acetone	Diffusion	Toluene	Slow Evaporation
$CHCl_3$/Hexane	Diffusion	Toluene/Pentane	Vapor Diffusion
$CHCl_3$/Hexane	Vapor Diffusion	Toluene/Hexane	Vapor Diffusion
$CHCl_3$/MeOH	Diffusion	Toluene/AcN	Vapor Diffusion
$CHCl_3$/EtOH	Diffusion	Chlorobenzene	Slow Evaporation
$CHCl_3$/ Et_2O	Diffusion	Chlorobenz./P. Ether	Vapor Diffusion
$CHCl_3$/ Et_2O	Vapor Diffusion	Chlorobenzene/MeOH	Diffusion

3. X-ray structure analysis

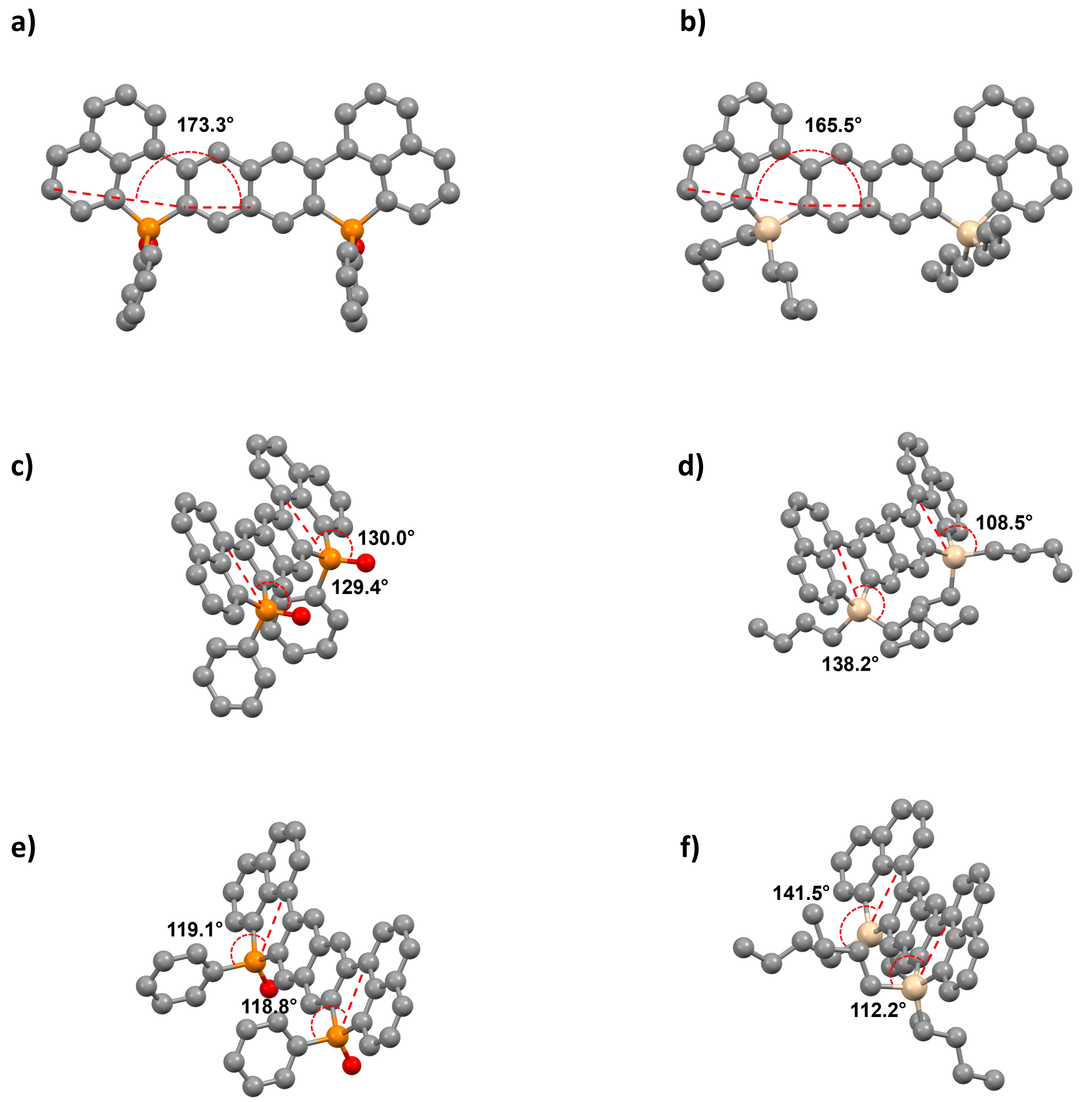

Figure S1. X-ray structure of (a, c, e) **1**[S1] and (b, d, f) **2** with the indicated angles. Hydrogens have been omitted for clarity.

Table S2. Selected crystallographic data of compound **2**.

Parameter	Value
**Empirical formula**	$C_{46}H_{52}Si_2$
**Molecular weight**	661.05 g/mol
**Temperature**	296.15 K
**Wavelength**	0.71073 Å
**Crystal system**	Triclinic
**Space group**	*P*-1
**Z**	4
**Unit cell dimensions**	a = 9.869(6) Å; α = 82.407(10) °
	b = 12.442(7) Å; β = 81.941(10) °
	c = 32.249(19) Å; γ = 74.764(8) °
**Volume**	3764(4) $Å^3$
**Density (calculated)**	1.167 g/cm^3
**Absorption coefficient**	0.126 mm^{-1}
**Crystal shape**	Needle
**Crystal size**	0.25 mm x 0.04 mm x 0.03 mm
**Crystal color**	Colorless
**Theta range for data collection**	3.41 – 44.9 °
**Index ranges**	$-10 \leq h \leq 10$, $-13 \leq k \leq 13$, $-34 \leq l \leq 31$
**Reflections collected**	20144
**Independent reflections**	9659 (R_{int} = 0.0811)
**Absorption correction**	Semi-empirical from equivalents
**Refinement method**	Full-matrix least-squares on F^2
**Data / restraints / parameters**	9659 / 1331 / 874
**Goodness-of-fit on F^2**	1.083
**Final R indexes [I>2σ(I)]**	R_1 = 0.1091, wR_2 = 0.2711
**Largest diff. peak and hole**	0.55 / –0.59 $e^-Å^{-3}$

4. DFT calculations

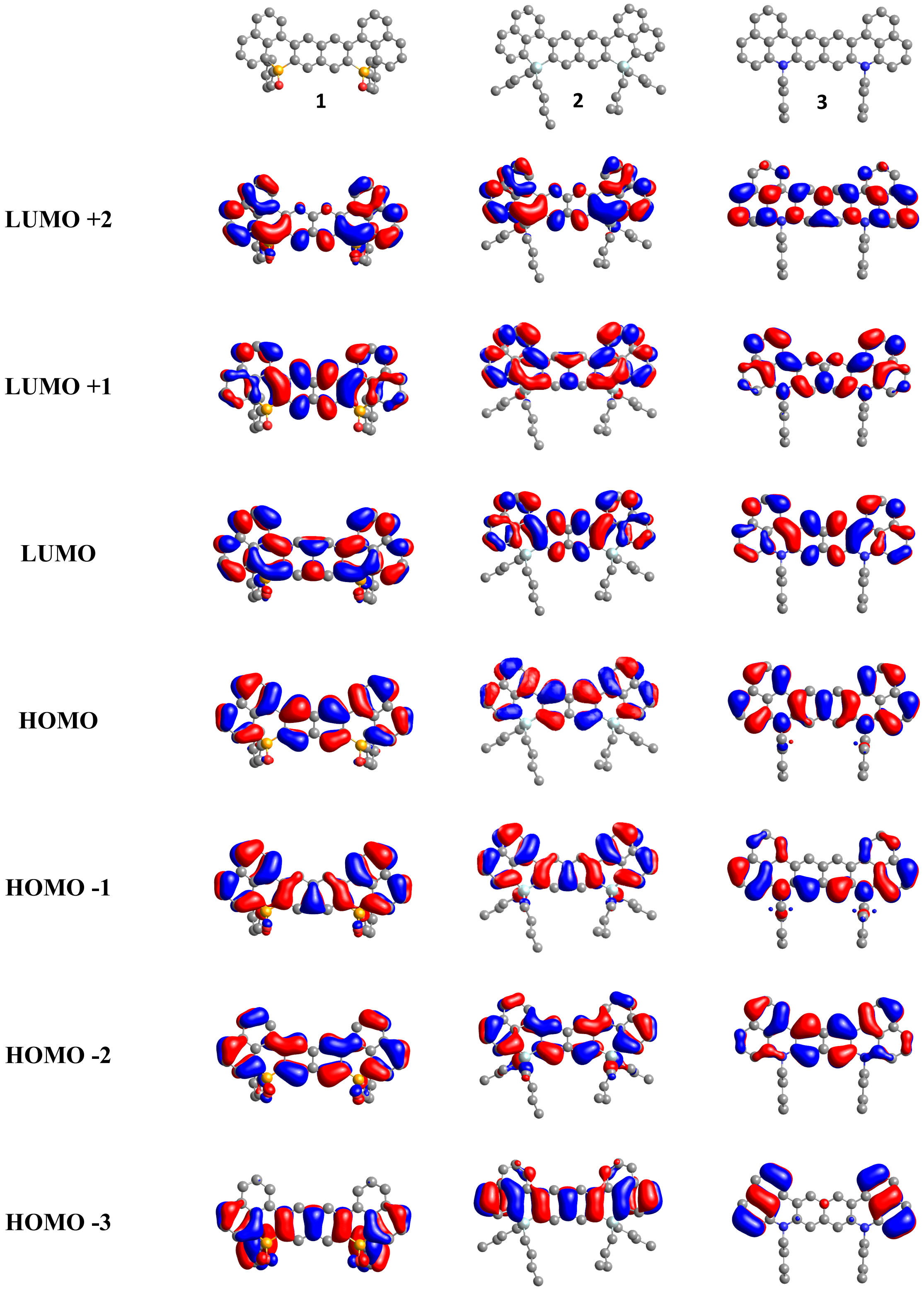

Figure S2. Most relevant molecular orbitals of compounds **1**, **2**, and **3** computed by DFT calculations at the B3LYP/6-311+G(d) level of theory.

Table S3. TD-DFT values obtained at the B3LYP/6-311+G(d) level of theory.

Compound	Transition	Oscillator Strength	Abs (nm)
**1**	HOMO → LUMO	0.8603	448.42
	HOMO → LUMO +1	0.0199	416.11
	HOMO -1 → LUMO +1	0.7952	395.71
**2**	HOMO → LUMO	0.9379	449.43
	HOMO -1 →LUMO<br>HOMO →LUMO +1	0.0157	414.56
	HOMO -1 → LUMO<br>HOMO -1 → LUMO+1<br>HOMO → LUMO+1	0.228	392.63
**3**	HOMO → LUMO	1.1092	576.77
	HOMO -1 → LUMO	0.0442	477.45
	HOMO -2 → LUMO<br>HOMO → LUMO +1<br>HOMO → LUMO +2	0.2288	392.63

Table S4. Orbital energies in eV of compounds **1-3** computed by DFT calculations at the B3LYP/6-311+G(d) level of theory.

	**1**	**2**	**3**
**LUMO +2**	-1.938	-1.404	-0.842
**LUMO +1**	-2.363	-1.925	-1.357
**LUMO**	-2.422	-1.934	-2.092
**HOMO**	-6.049	-5.717	-4.896
**HOMO -1**	-6.321	-5.866	-5.324
**HOMO -2**	-7.008	-6.438	-5.733
**HOMO -3**	-7.148	-6.905	-6.966
**GAP**	3.627	3.783	2.804

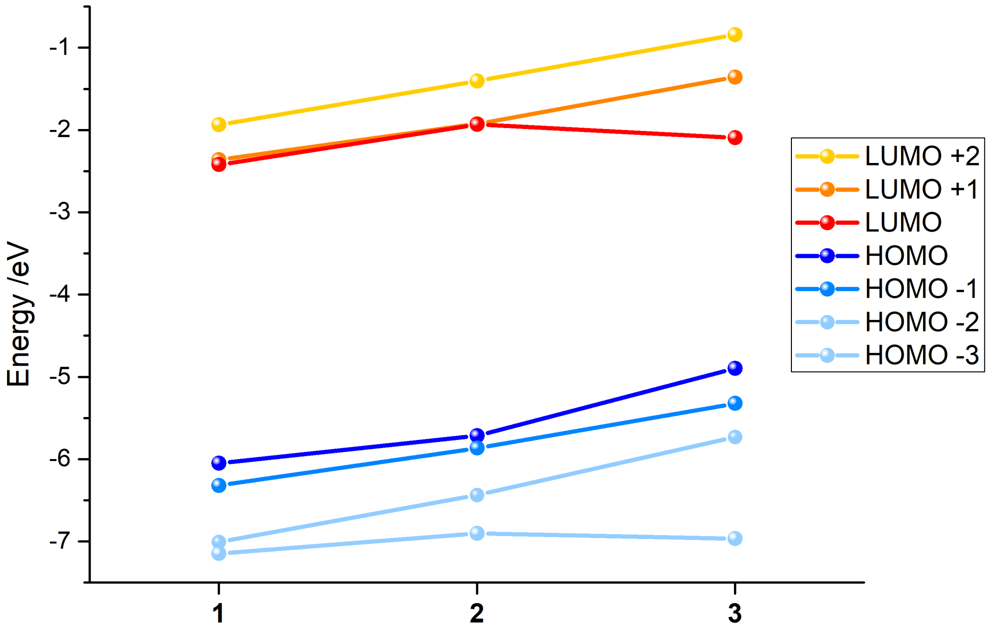

Figure S3. Energy of the most relevant molecular orbitals of compounds **1**, **2**, and **3** computed by DFT calculations at the B3LYP/6-311+G(d) level of theory.

5. Electrochemical investigations of compounds 1, 2, and 3

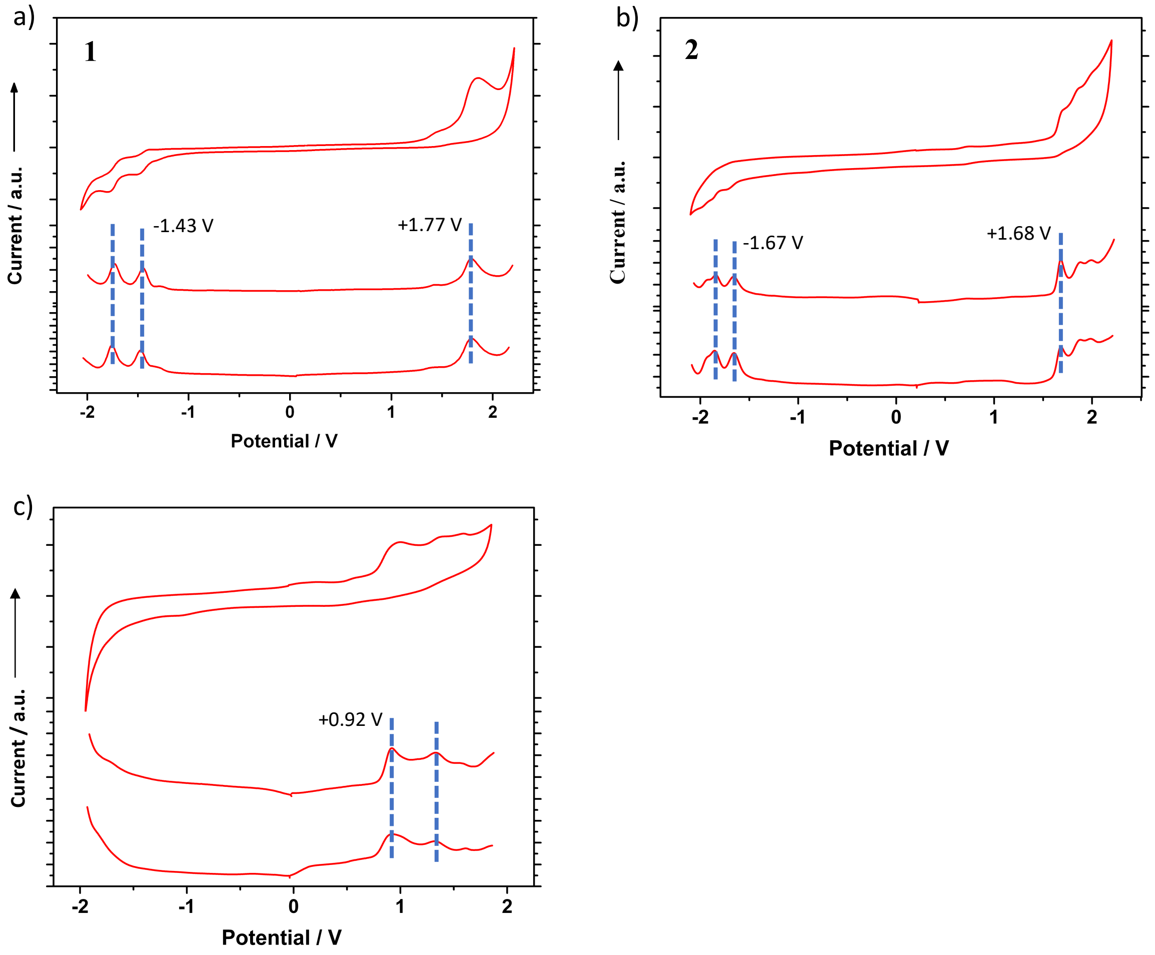

Figure S4. Voltammograms of a) compound **1**,[S1] b) compound **2**, and c) compound **3** recorded from acetonitrile solutions containing tetrabutylammonium hexafluorophosphate as the supporting electrolyte. Measurements were performed using a glassy carbon working electrode, a platinum wire counter electrode, and a silver wire pseudo-reference electrode at a scan rate of 100 mV s^{-1}. For each compound, the top graph shows cyclic voltammetry (CV), which provides information on the reversibility and stability of the redox processes by observing the shape and peak separation of the voltammogram. The middle and bottom graphs show differential pulse voltammetry (DPV) and square wave voltammetry (SWV), respectively. These latter two techniques are more sensitive and offer higher resolution, allowing for more accurate identification and precise determination of the redox peak potentials, especially when peaks are closely spaced or when current responses are low. Dashed lines indicate the correlation of the peaks across the different techniques. All values were obtained as an average of three independent measurements. Numbers on the graphs indicate the lowest redox processes (see Table S5 for all values).

Table S5. Redox properties of compounds **1**,[S1] **2**, and **3**.

Compound	Red. 3 $E_{1/2}$ (V)	Red. 2 $E_{1/2}$ (V)	Red. 1 $E_{1/2}$ (V)	Ox. 1 $E_{1/2}$ (V)	Ox. 2 $E_{1/2}$ (V)	Ox. 3 $E_{1/2}$ (V)
**1**[S1]		-1.71	-1.43	1.77		
**2**	-1.97	-1.85	-1.66	1.68	1.88	1.98
**3**				0.92	1.32	1.58

6. Steady-state spectroscopy of compounds 1, 2, and 3

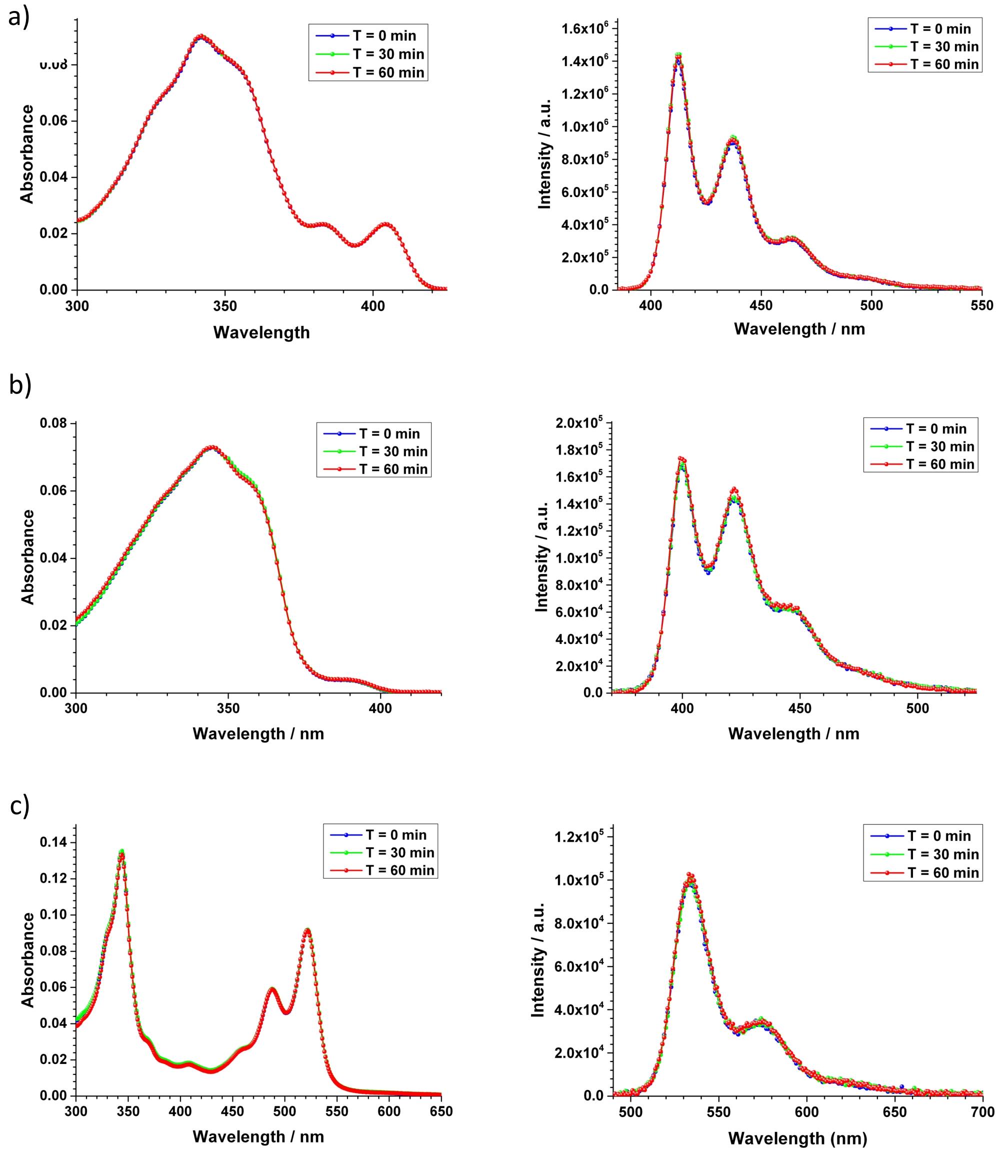

Figure S5. Stability tests. Absorption and emission spectra of: a) compound **1**, b) compound **2**, and c) compound **3** recorded at 0, 30 and 60 min from DCM solutions.

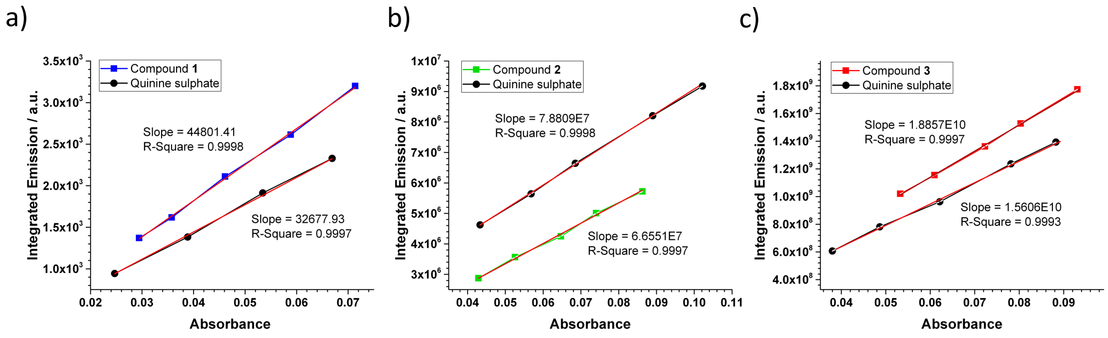

Figure S6. Representations of integrated emission vs. absorbance used for the calculations of the fluorescence quantum yields of a) compound **1**, b) compound **2**, and c) compound **3** from DCM solutions.

<u>Parameters used for the calculation of the fluorescence quantum yields</u>:

Formula: $\Phi_x = \Phi_{st}\ (Grad_x/Grad_{st})\ (n_x^2/n_{st}^2)$; $\Phi_{st} = 0.54$; $n_x = 1.424$; $n_{st} = 1.133$

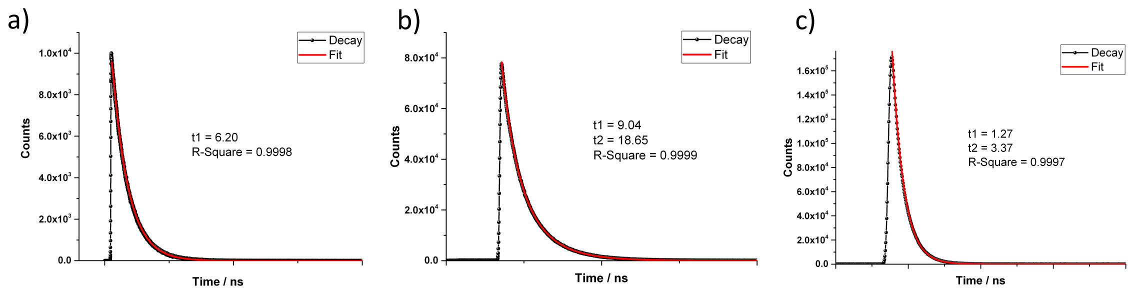

Figure S7. Representations of fluorescence decays with their fitting values of a) compound **1**, b) compound **2**, and c) compound **3** from DCM solutions.

Table S6. Selected spectroscopic data from compounds **1**,[S1] **2**, and **3**.

	**1**	**2**	**3**
**Absorp. λ_{max} (nm)**[a]	343, 405	343, 358 (sh), 390	343, 487, 522
**Emission λ_{max} (nm)**[b]	415, 438, 463	400, 423, 446, 480 (sh)	535, 579, 626
**log ε**[c]	4.4	4.7	4.7
**τ (ns)**[d]	6.2	9.04, 18.65	1.27, 3.37
**Φ (%)**[e]	84	50	74

[a] Absorption maxima recorded from DCM solutions. [b] Emission maxima recorded from DCM solutions. [c] Molar extinction coefficient of the absorption maxima. [d] Fluorescence lifetimes. [e] Fluorescence quantum yields relative to quinine sulfate in 0.1M H_2SO_4, $\Phi = 0.54$.

7. NMR data

¹H NMR (400 MHz, Chloroform-*d*) of compound **2**

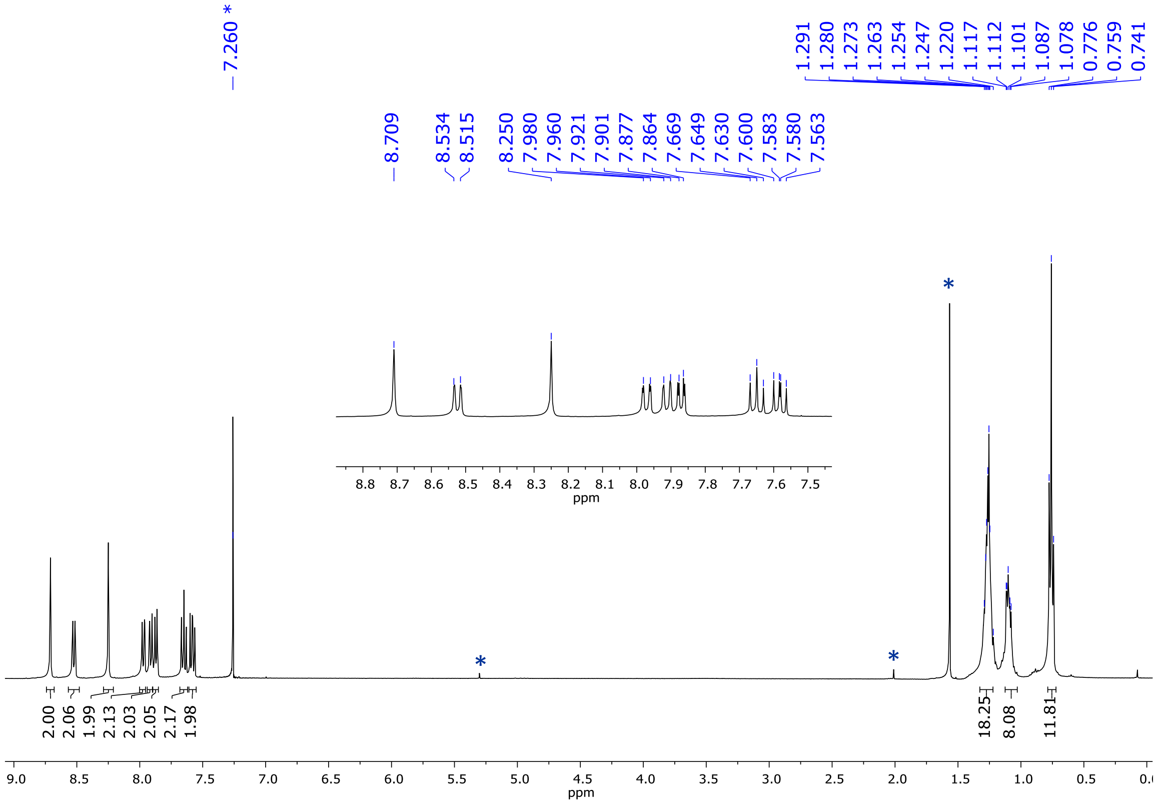

$^{13}C\{^1H\}$ NMR (101 MHz, Chloroform-*d*) of compound **2**

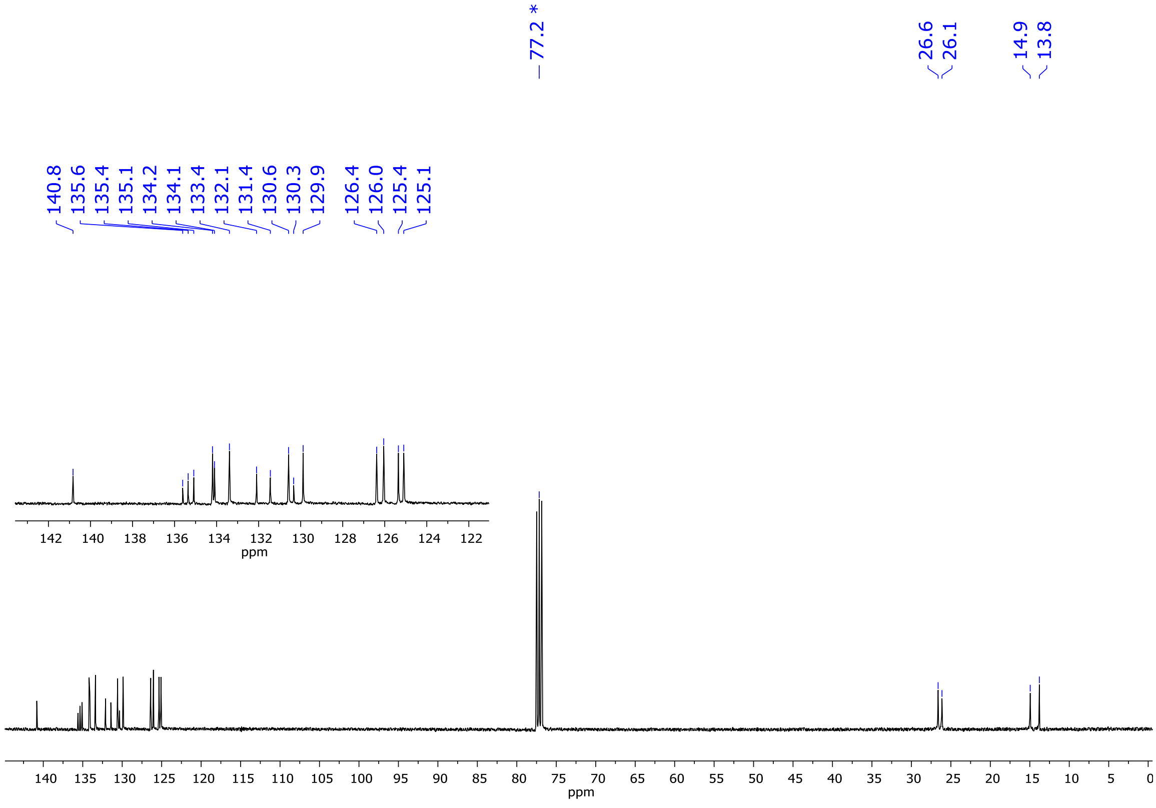

^{1}H-^{1}H COSY (400 MHz, Chloroform-*d*) of compound **2**

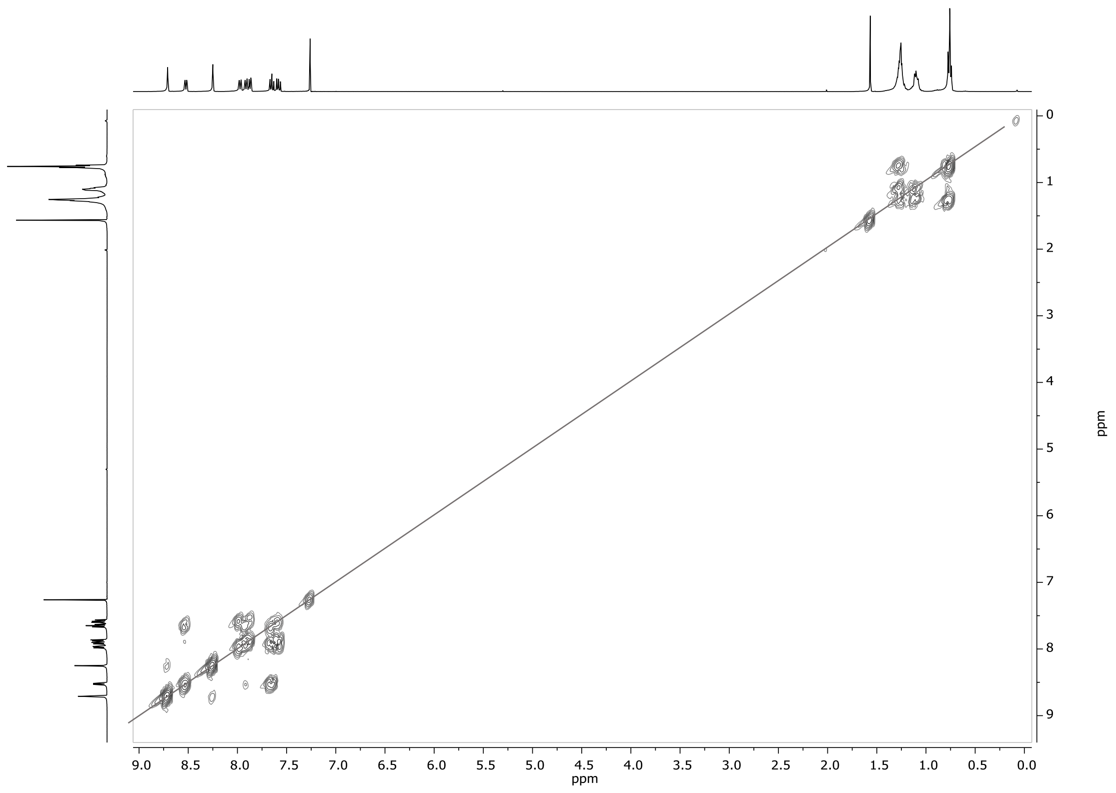

^{1}H NMR (400 MHz, Chloroform-*d*) of compound **3**

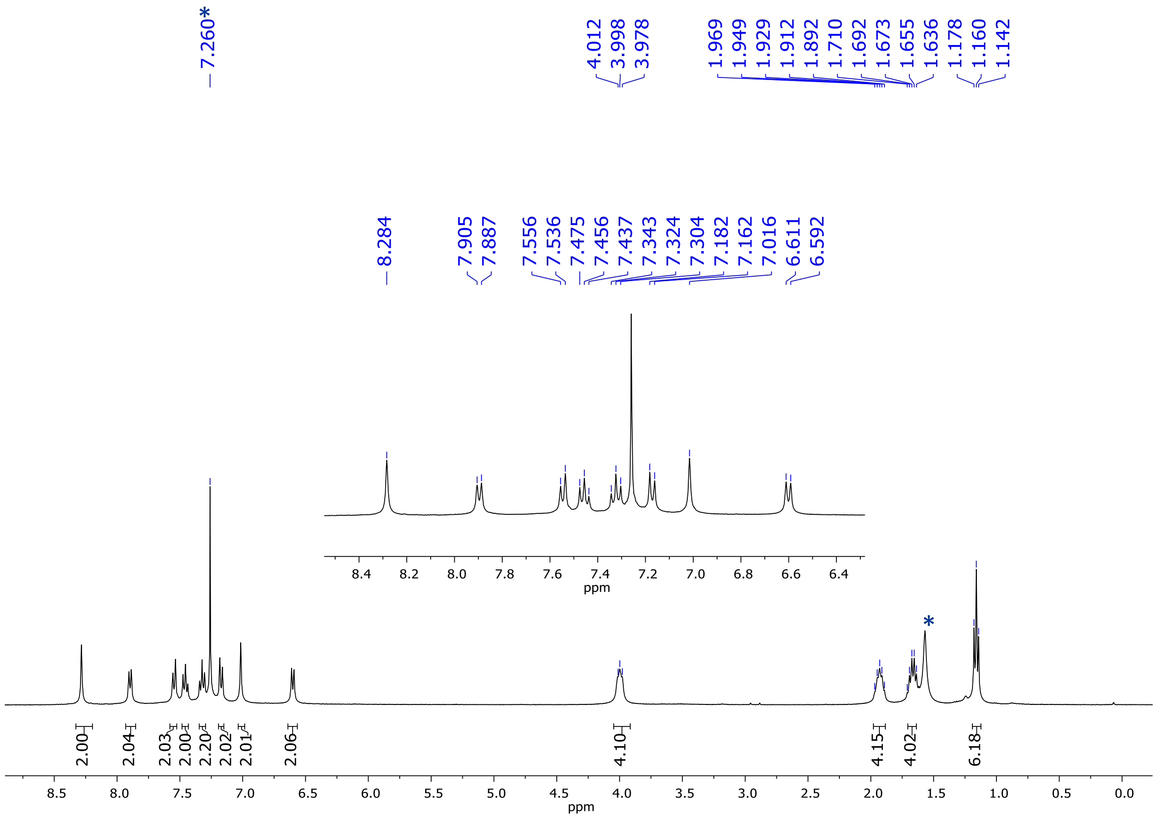

$^{13}C\{^{1}H\}$ NMR(101 MHz, Chloroform-*d*) of compound **3**

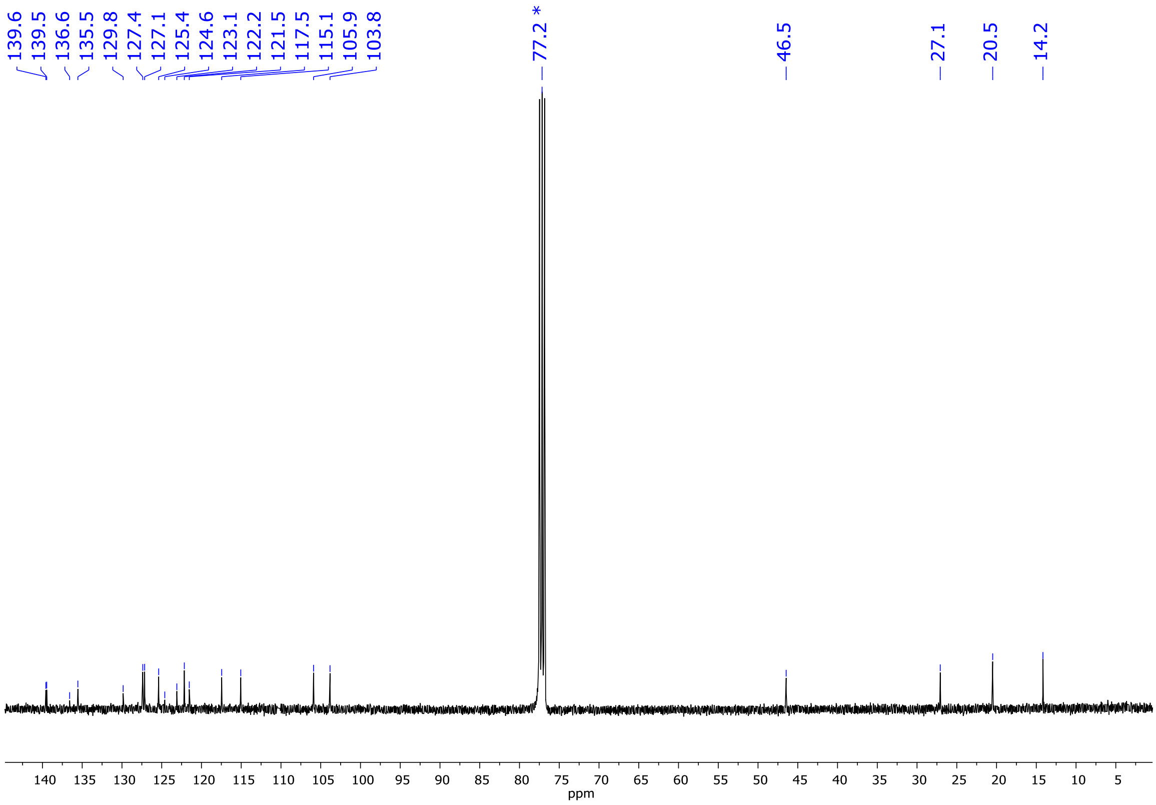

^{1}H-^{1}H COSY (400 MHz, Chloroform-*d*) of compound **3**

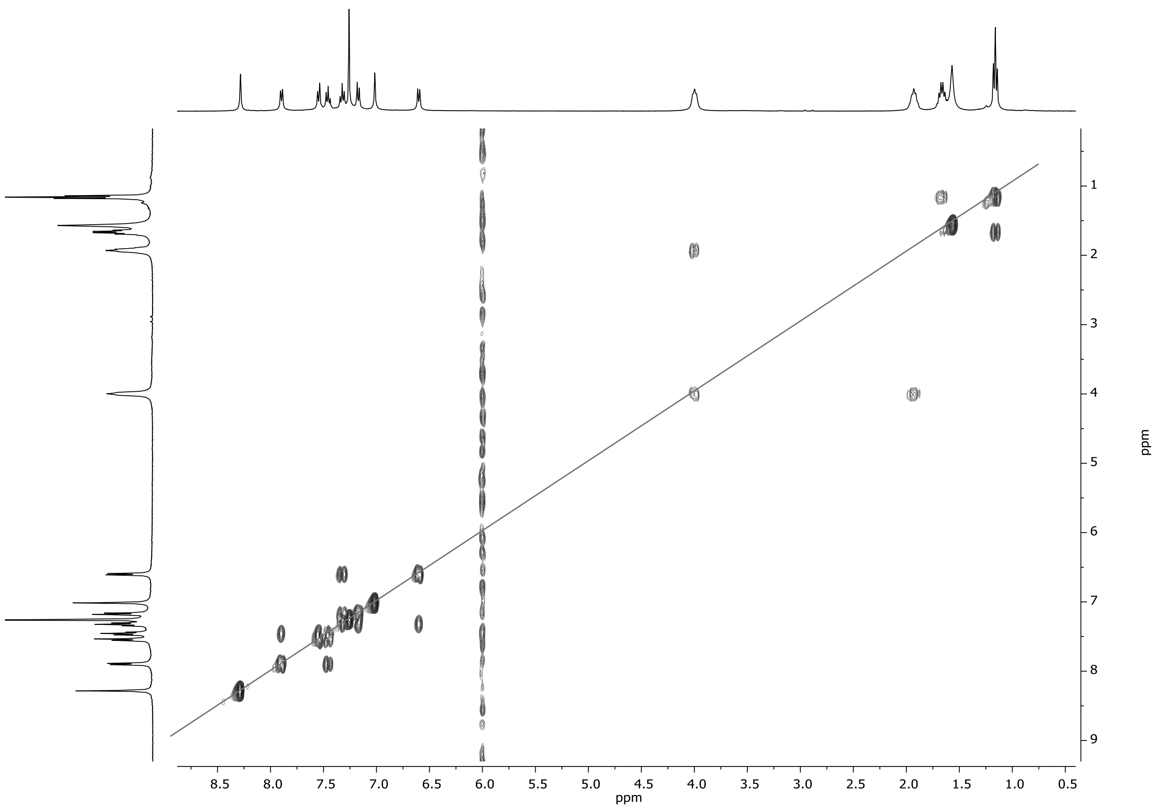

8. Literature

S1. P. Hindenberg, M. Busch, A. Paul, M. Bernhardt, P. Gemessy, F. Rominger and C. Romero-Nieto, *Angew. Chem. Int. Ed.* **2018**, *57*, 15157–15161.

S2. Program SADABS 2008/1 for absorption correction; G. M. Sheldrick, Bruker Analytical X-ray-Division, Madison, Wisconsin 2012.

S3. Software package SHELXTL 2008/4 for structure solution and refinement; G.M. Sheldrick, *Acta Cryst.* 2008, **A64**, 112.

S4. Gaussian 16, Revision C.01, M. J. Frisch, G. W. Trucks, H. B. Schlegel, G. E. Scuseria, M. A. Robb, J. R. Cheeseman, G. Scalmani, V. Barone, G. A. Petersson, H. Nakatsuji, X. Li, M. Caricato, A. V. Marenich, J. Bloino, B. G. Janesko, R. Gomperts, B. Mennucci, H. P. Hratchian, J. V. Ortiz, A. F. Izmaylov, J. L. Sonnenberg, D. Williams-Young, F. Ding, F. Lipparini, F. Egidi, J. Goings, B. Peng, A. Petrone, T. Henderson, D. Ranasinghe, V. G. Zakrzewski, J. Gao, N. Rega, G. Zheng, W. Liang, M. Hada, M. Ehara, K. Toyota, R. Fukuda, J. Hasegawa, M. Ishida, T. Nakajima, Y. Honda, O. Kitao, H. Nakai, T. Vreven, K. Throssell, J. A. Montgomery, Jr., J. E. Peralta, F. Ogliaro, M. J. Bearpark, J. J. Heyd, E. N. Brothers, K. N. Kudin, V. N. Staroverov, T. A. Keith, R. Kobayashi, J. Normand, K. Raghavachari, A. P. Rendell, J. C. Burant, S. S. Iyengar, J. Tomasi, M. Cossi, J. M. Millam, M. Klene, C. Adamo, R. Cammi, J. W. Ochterski, R. L. Martin, K. Morokuma, O. Farkas, J. B. Foresman, and D. J. Fox, Gaussian, Inc., Wallingford CT, 2016.

S5. W. H. Melhuish, *J. Phys. Chem.* **1961**, *65*, 229.
